# Evolution of *hydra,* a Recently Evolved Testis-Expressed Gene with Nine Alternative First Exons in Drosophila melanogaster

**DOI:** 10.1371/journal.pgen.0030107

**Published:** 2007-07-06

**Authors:** Shou-Tao Chen, Hsin-Chien Cheng, Daniel A Barbash, Hsiao-Pei Yang

**Affiliations:** 1 Faculty of Life Sciences and Institute of Genome Sciences, National Yang-Ming University, Taipei, Taiwan, Republic of China; 2 Department of Molecular Biology and Genetics, Cornell University, Ithaca, New York, United States of America; Fred Hutchinson Cancer Research Center, United States of America

## Abstract

We describe here the *Drosophila* gene *hydra* that appears to have originated de novo in the *melanogaster* subgroup and subsequently evolved in both structure and expression level in Drosophila melanogaster and its sibling species. *D. melanogaster hydra* encodes a predicted protein of ~300 amino acids with no apparent similarity to any previously known proteins. The syntenic region flanking *hydra* on both sides is found in both D. ananassae and *D. pseudoobscura,* but *hydra* is found only in *melanogaster* subgroup species, suggesting that it originated less than ~13 million y ago. Exon 1 of *hydra* has undergone recurrent duplications, leading to the formation of nine tandem alternative exon 1s in *D. melanogaster.* Seven of these alternative exons are flanked on their 3′ side by the transposon *DINE-1* (*Drosophila* interspersed element-1). We demonstrate that at least four of the nine duplicated exon 1s can function as alternative transcription start sites. The entire *hydra* locus has also duplicated in D. simulans and D. sechellia. *D. melanogaster hydra* is expressed most intensely in the proximal testis, suggesting a role in late-stage spermatogenesis. The coding region of *hydra* has a relatively high Ka/Ks ratio between species, but the ratio is less than 1 in all comparisons, suggesting that *hydra* is subject to functional constraint. Analysis of sequence polymorphism and divergence of *hydra* shows that it has evolved under positive selection in the lineage leading to *D. melanogaster.* The dramatic structural changes surrounding the first exons do not affect the tissue specificity of gene expression: *hydra* is expressed predominantly in the testes in *D. melanogaster, D. simulans,* and *D. yakuba.* However, we have found that expression level changed dramatically (~ >20-fold) between D. melanogaster and *D. simulans.* While *hydra* initially evolved in the absence of nearby transposable element insertions, we suggest that the subsequent accumulation of repetitive sequences in the *hydra* region may have contributed to structural and expression-level evolution by inducing rearrangements and causing local heterochromatinization. Our analysis further shows that recurrent evolution of both gene structure and expression level may be characteristics of newly evolved genes. We also suggest that late-stage spermatogenesis is the functional target for newly evolved and rapidly evolving male-specific genes.

## Author Summary

Similar groups of animals have similar numbers of genes, but not all of these genes are the same. While some genes are highly conserved and can be easily and uniquely identified in species ranging from yeast to plants to humans, other genes are sometimes found in only a small number or even in a single species. Such newly evolved genes may help produce traits that make species unique. We describe here a newly evolved gene called *hydra* that occurs only in a small subgroup of *Drosophila* species. *hydra* is expressed in the testes, suggesting that it may have a function in male fertility. *hydra* has evolved significantly in its structure and protein-coding sequence among species. The authors named the gene *hydra* after the nine-headed monster slain by Hercules because in one species, *Drosophila melanogaster, hydra* has nine potential alternative first exons. Perhaps because of this or other structural changes, the level of RNA made by *hydra* differs significantly between one pair of species. This analysis reveals that newly created genes may evolve rapidly in sequence, structure, and expression level.

## Introduction

Much of the genetic novelty that accompanies speciation and organismal evolution is driven by reutilization of pre-existing genetic information. In an influential essay, Francois Jacob likened evolution to a process of tinkering [1]. After the primordial evolution of truly new macromolecules and mechanisms of replication, Jacob suggested that much of phenotypic novelty arises from reusing, recombining, and altering the function of available genes.

This view of evolution is strongly supported by studies of new-gene evolution. The vast majority of newly evolved genes can be attributed to duplication of pre-existing genes. These duplication events can range from single-gene events to duplications of entire genomes [2,3]. Much of protein evolution also conforms to Jacob's view, as new proteins are often generated from shuffling pre-existing protein domains [4].

The tinkering view of evolution does not rule out the occasional generation of novel protein sequences. One question then is whether and by what mechanisms such novel proteins evolve. Presumably novel proteins derive from noncoding sequences that acquire appropriate transcriptional and translational regulatory sequences. Experimental evolution studies suggest that random sequences of proteins can acquire biological functions at frequencies that are, surprisingly, greater than miniscule [5]. A recent study reported the exciting finding of several such candidate de novo genes in *Drosophila* that may be functional, based on RNA expression analysis [6]. A large number of new exons have also been identified in rodents that appear to have derived from incorporation of intronic sequences into mRNAs [7].

A large fraction of eukaryotic genomes is composed of transposable elements (TEs). Because the TE component of genomes can evolve rapidly, TEs have a major impact on genome evolution [8–10]. TEs are widely considered to be selfish parasites that are deleterious to their host's fitness [11], and most are likely eliminated quickly by natural selection. However, accumulating evidence has also shown that TEs can serve as an important source of genetic variation and genomic novelty [8,12–14].

TEs can generate genetic novelty by at least five different mechanisms. First, the reverse transcriptase enzyme from retrotransposons can generate duplicated retroposed genes [15–17] or generate chimeric fusion genes [15,18–21]. Second, TEs can change expression patterns of adjacent genes by providing novel regulatory elements or by disrupting host gene regulatory functions [14,22–26]. Third, coding regions from TEs such as envelope proteins and transposase enzymes can be incorporated by the host species to modify or even create new host protein-coding genes [13,27]. Fourth, TEs can contribute to noncoding regions (untranslated regions [UTRs]) of genes [12,24,28–30]. Fifth, TE insertions create multiple highly homologous sites throughout the genome that can cause duplications by ectopic recombination [9,31].

Comparisons between genomes have proven to be a powerful way to identify new genes [32] and to reconstruct the early history of new-gene evolution [2]. This approach has provided new data for models and mechanisms leading to new-gene evolution [6,20,33–35].

Such a comparative approach is used here to investigate the evolutionary history of a recently evolved gene in *Drosophila.* We report the characterization of the gene *hydra,* located in the pericentric region of the X chromosome in *D. melanogaster. hydra* was originally reported as predicted gene *CG1338* [36]. We show that *hydra* originated in the *melanogaster* subgroup of *Drosophila* and subsequently experienced dramatic changes in its gene structure, including the formation of multiple alternative first exons, most of which are associated with the transposon *DINE-1* (*Drosophila* interspersed element-1)*.* We also investigate the history and impact of these gene-structure changes at both the DNA sequence and RNA expression levels by comparison among species of the *melanogaster* subgroup*.* We suggest that new genes will tend to evolve rapidly in coding sequence (CDS), gene structure, and gene-expression level. We also suggest that TE accumulation can cause local heterochromatinization that can in turn have a major impact on the evolution of gene structure and gene expression.

## Results

### The *hydra* Gene Is Restricted to the *melanogaster* Subgroup

**Figure 1:**
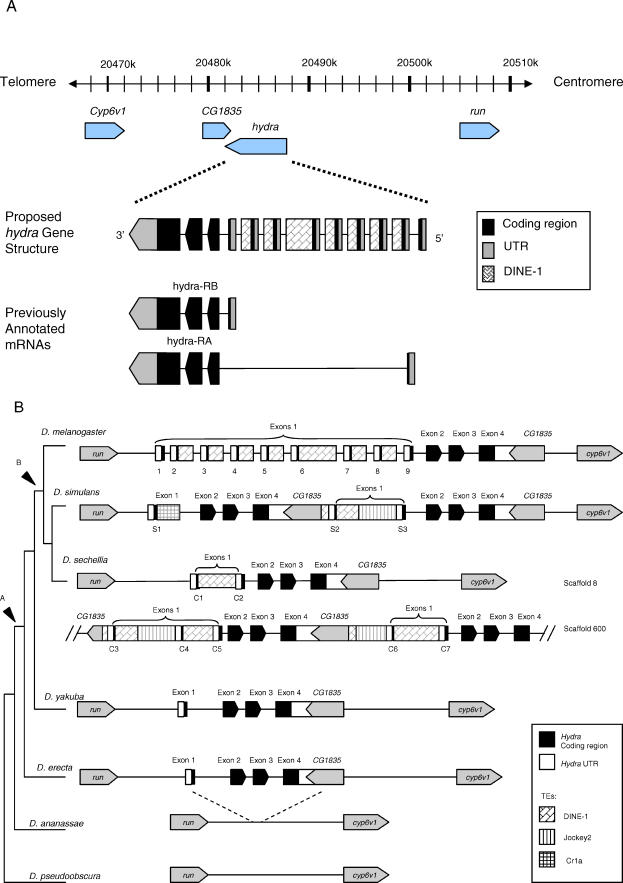
Evolution of *hydra* in *Drosophila* Species (A) The *hydra* region of the X-chromosome of *D. melanogaster,* based on FlyBase genome browser release 4.3. The *hydra* gene was previously annotated as producing two alternative transcripts, RA and RB, derived from alternative exon 1s. The proposed annotation of seven additional exon 1s is based on evidence presented here. (B) Evolution of *hydra* region and *hydra* gene structure in seven Drosophila species. *hydra* and the flanking gene *CG1835* are located in a recently expanded region between *run* and *cyp6v1. hydra* originated in the common ancestor of the *melanogaster* subgroup (arrow A). In *D. melanogaster,* this region between *run* and *cyp6v1* is ~32 kb (10 kb from the 3′ end of *hydra* to *cyp6v1* and 17 kb from the 5′ end of *hydra* to *run*), but is only ~26 kb apart in D. ananassae and *D. pseudoobscura,* where both *hydra* and *CG1835* are missing. *hydra* has gone through multiple cycles of duplication and rearrangement in D. melanogaster and its sibling species, and accumulated insertions of the transposon *DINE-1* and other repetitive sequences (arrow B). *CG1835* is on the opposite strand from all other genes, as indicated by its leftward-pointing arrow. Three copies of *hydra* are found on two unlinked scaffolds in *D. sechellia.* Note that the distances are not to scale.

*CG1338* is located near the pericentric region of the X chromosome in D. melanogaster (cytological region 19E1). We first became interested in *CG1338* during a genome-wide analysis of the transposon *DINE-1* in the D. melanogaster genome. We present evidence below that *CG1338* contains nine duplicated first exons in D. melanogaster. Because of these nine duplicated exon 1s (see Figure 1A), we propose to rename this gene *hydra,* after the nine-headed monster slain by Hercules.

We found that the gene region surrounding *hydra* in D. melanogaster contains dense repeats of *DINE-1* (Figure 1A). *DINE-1,* also named *INE-1* or *DNAREP1_DM,* is a highly abundant transposon that is predominantly found in the heterochromatic regions of the D. melanogaster genome [37,38] and is believed to have invaded the *Drosophila* genome before the diversification of the *melanogaster* subgroup [37–40]. Further analysis showed that *hydra* is flanked by two blocks of tandem repeats, with duplicated fragments of *DINE-1* in the 5′ end and an undefined 500-bp repeat in the 3′ end. Using available genomic contigs from various *Drosophila* species (http://flybase.net/blast), we found that the ~7-kb region surrounding *hydra,* including the adjacent gene *CG1835,* only exists in species of the *melanogaster* subgroup, but not in other species outside of the subgroup. BLAST searches using unique sequences from *hydra* identified no similar sequences in other regions of these genomes. We suggest that *hydra* evolved recently in the species of the *melanogaster* subgroup (Figure 1B). This conclusion was confirmed by Southern hybridization under low stringency using *hydra* exons 2–4 from D. melanogaster as the probe with DNA extracted from *D. melanogaster, D. simulans, D. yakuba, D. erecta, D. ananassae, D. pseudoobscura,* and *D. virilis.* Hybridization signals were only detected in flies of the *melanogaster* subgroup (i.e., *D. melanogaster, D. simulans, D. yakuba, D. erecta*), but not in the other species (unpublished data). TBLASTN searches to the nonredundant (nr) or whole-genome shotgun (wgs) databases found no hits to non-*melanogaster* subgroup *Drosophila* species with E values < 0.001.

### Evolution of the Gene Structure of *hydra*

According to the Flybase genome annotation (http://www.flybase.org), *hydra* of D. melanogaster contains four exons, and two types of transcripts (type A and type B), which are transcribed using alternative first exons. Our analysis of the *hydra* gene region suggested that there are seven additional putative first exons similar to the two annotated first exons located in a region of dense *DINE-1s* in D. melanogaster (Figure 1A; Text S1). These seven additional potential first exons share similarity with the two annotated exon 1s in their CDSs (which are nine codons long) and in their 5′ upstream regions. The first of these duplicated exons may be nonfunctional because it creates a stop codon when spliced to exon 2, assuming that it has the same structure and splicing pattern as the other 8 exons. Alternatively, this first exon 1 may use a different 3′ splice site, although no such evidence was found in our 5′ rapid amplification of cDNA ends (RACE) analysis described below (Table 1).

**Table 1:**
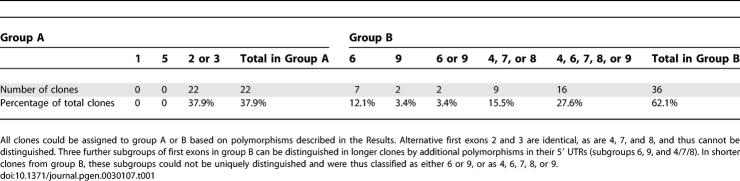
Relative Usage of Different Exon 1s of *hydra,* Determined by 5′ RACE

**Figure 2:**
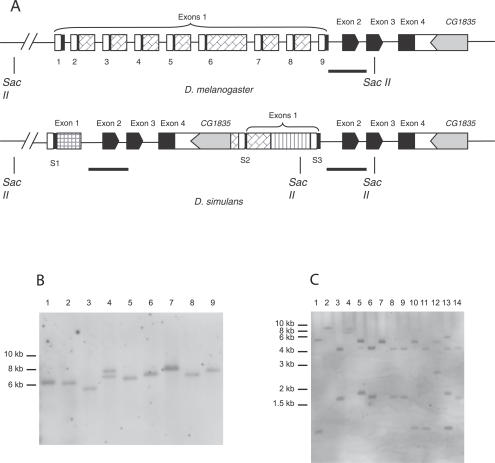
Length Variation of the Exon 1 Region of *hydra* in D. melanogaster (A) Location of the probes (thick black lines) and restriction enzyme SacII cutting sites in *hydra* of D. melanogaster (top) and *D. simulans* (bottom)*.* Note that the region homologous to the probe is duplicated in *D. simulans.* (B–C) Southern hybridization of genomic DNA extracted from multiple lines of D. melanogaster (B) and *D. simulans* (C)*. D. melanogaster* lines are all from Zimbabwe, Africa. D. simulans lines 1–4 are from California, United States; lines 5–7 are from Zimbabwe, Africa; and lines 8–14 are from Madagascar, Africa.

Our further analysis of multiple lines of D. melanogaster (and D. simulans) with Southern hybridization detected substantial length variation in the gene region, presumably due to length variation in the exon 1 region (Figure 2). Although we have not determined the sequence structure of these alleles, the length variation observed is consistent with the hypothesis that the number of alternative first exons varies in *D. melanogaster.* In contrast, there is only a single exon 1 in the orthologous region of *hydra* from the species D. yakuba and D. erecta (Texts S5 and S6), and there is no length polymorphism in this region in D. yakuba (unpublished data). This suggests that the dramatic gene structure change by duplication of exon 1 in *hydra* occurred within ~5–13 million y, after the divergence of D. melanogaster and D. yakuba [41,42].

**Figure 3:**
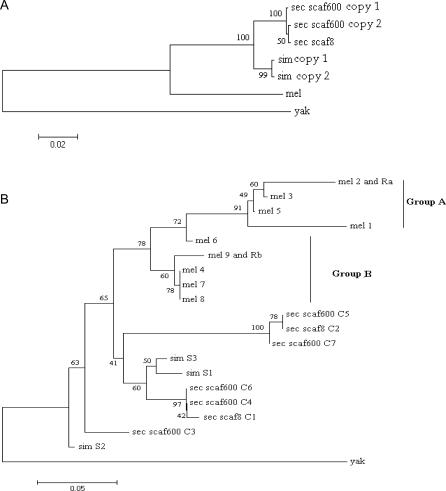
Phylogenetic Trees of *hydra* Trees are based on (A) exon 2–4 sequences and (B) exon 1, including putative 5′ UTR sequences. The trees were built using the neighbor-joining method according to the Jukes-Cantor substitution model. Branch lengths are proportional to the number of nucleotide substitutions. Percentage bootstrap values are shown at each node (500 replicates). In (B), sequences mel 1–9 correspond to the alternative exon 1s of D. melanogaster shown in Figure 1B. Sequences Ra and Rb are from the annotated transcripts of *D. melanogaster hydra* in FlyBase. D. simulans and *D. sechellia* exon 1s are labeled as shown in Figure 1B. Note that mel 6 is part of group B but clusters with group A, presumably because of having a divergent 5′ UTR.

By comparing the orthologous sequences of *hydra* using the released assembled genomic sequences of D. simulans and *D. sechellia,* we found that this region has undergone local duplications in both species (Figure 1B; Texts S2–S4). Determining the precise structure of the *hydra* region in D. sechellia will require further sequence analysis due to low sequence coverage of this region. We have, however, confirmed by Southern analysis that D. simulans does have two copies of *hydra* (Figure 2C). In *D. simulans,* we find that one of these copies has two potential exon 1s. In *D. sechellia,* we identified two unlinked contigs containing *hydra*-homologous sequences (Figure 1B). Scaffold 8 contains the syntenic genes *run* and *cyp6v1* flanking a single copy of *hydra.* This copy of *hydra* contains two putative exon 1s. Scaffold 600 contains two tandem copies of *hydra,* each of which contains two or three putative exon 1s. These data suggest the possibility that D. sechellia has three copies of *hydra,* although further analysis will be required to determine the relative locations of these apparent duplicated copies that are on different scaffolds. Despite this uncertainty, we were able to investigate the phylogenetic relationship of these copies of *hydra.* Using either the sequence between exons 2–4, inclusive (Figure 3A; Text S7) or of intron 1 (unpublished data), we obtained similar phylogenetic trees that suggest that *hydra* duplicated independently in D. simulans and *D. sechellia.* Because both gene regions produce the same phylogeny and these sequences span over 1,300 bp in all species, we consider it unlikely that this phylogenetic signal of independent duplications could be due to gene conversion.

Upon further investigation of the intronic sequences next to exon 1, we found that seven out of the nine duplicated exon 1s in D. melanogaster and several of the duplicated exon 1s in both D. simulans and D. sechellia are immediately followed by *DINE-1* sequences, while in other species of the *melanogaster* subgroup, including D. yakuba and *D. erecta,* there are no *DINE-1s* in this region. We thought it likely for two reasons that insertion of *DINE-1* adjacent to *hydra* exon 1 occurred in the common ancestor of *D. melanogaster, D. simulans,* and *D. sechellia.* First, it seems highly unlikely that a TE would insert independently at the same location in different species. Second, *DINE-1* is thought to have been active and then to have become transpositionally inactive before these species diverged [38,40].

To test this hypothesis and to understand the evolutionary history and mechanism that resulted in the exon 1 duplications of *hydra,* we performed phylogenetic analysis of all the available exon 1s (Figure 3B; Text S8). We found that D. simulans S2 and D. sechellia C3 are outgroups to all other exon 1s. Both of these exons are flanked by *DINE-1* sequence, which is consistent with our above suggestion that *DINE-1* insertion in this region is ancestral (at time B in Figure 1B). If this inference of the ancestral state of exon 1 in these three species is correct, it is interesting to note that *DINE-1* does not flank every exon 1. Our data would then suggest that either *DINE-1* was lost next to some duplicated exon 1s or that some exon 1s duplicated without duplicating the adjacent *DINE-1.*

The relationships among the remaining D. simulans and D. sechellia exon 1s suggest that exon 1 duplicated before the speciation of D. simulans and D. sechellia to generate the exon 1 groups S1/S3 and C1/C4/C6, with another duplication event generating exon 1 group C2/C5/C7 (Figure 3B). Both this tree and the one made using the rest of the locus (Figure 3A) suggest that after these exon 1 duplications, the entire locus duplicated independently in D. simulans and *D. sechellia.*

Figure 3B also suggests that exon 1 duplicated independently in *D. melanogaster.* Based on the sequence similarity of predicted exon 1 sequences, the duplicated first exons of D. melanogaster can be divided into three groups: group A, containing the first, second, third, and fifth duplicated exon 1s; group B, containing the fourth, seventh, eighth, and ninth duplicated exon 1s; and group C, containing the sixth duplicated exon 1. The first and the ninth exon 1s are the previously annotated exon 1s for transcript types RA and RB, respectively. Our phylogenetic analysis showed that exon 1s adjacent to each other are generally more closely related (Figure 3B). These results suggest that both the exon 1s and their flanking *DINE-1*s were duplicated together, and that unequal crossing-over is the major mechanism generating the tandemly duplicated exons.

### *hydra* Is Expressed Predominantly in Adult Testes

**Figure 4:**
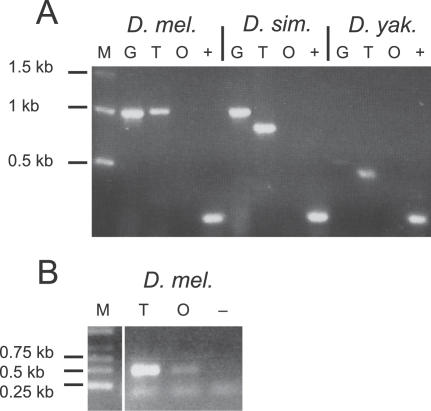
Expression of *hydra* detected by RT-PCR (A) *D. melanogaster, D. simulans,* and *D. yakuba.* (B) Canton S line of *D. melanogaster.* M, DNA kb size marker; G, genomic DNA; T, adult testis RNA; O, RNA from whole body of males with testis removed; +, positive control for cDNA O using primers for the gene *GAPDH*; −, negative PCR control. RNA was extracted from strain ORC of D. melanogaster (A), California strain w54 of *D. simulans,* and Tai18E2 of D. yakuba. Note that expression of *hydra* in D. melanogaster is specific to testis in the ORC strain (A), but slight expression was detected in other tissues in the Canton S strain (B).

**Figure 5:**
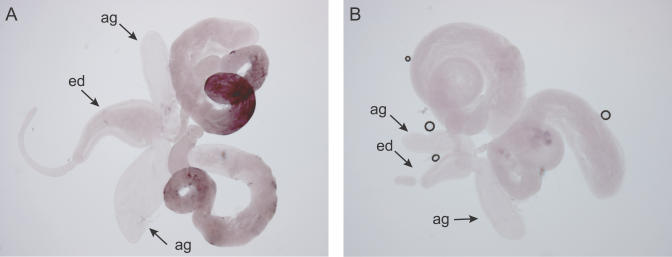
In Situ Hybridization of DIG-Labeled Antisense RNA (A) and Sense Control RNA (B) of *hydra* to Testes of D. melanogaster Expression is strongest in the proximal region of testes. ag, accessory glands; ed, ejaculatory duct.

RT-PCR analysis demonstrated that *hydra* is expressed in adult male testes in all three species tested: *D. melanogaster, D. simulans,* and D. yakuba (Figure 4). This result demonstrates that the expression pattern of *hydra* is conserved despite having a very different gene structure in these species. In one out of two stocks of D. melanogaster that we examined, we also detected a low level of expression in the male carcass from which testes were removed (Figure 4B). This expression pattern is consistent with D. melanogaster EST data: among ten ESTs in GenBank that match *hydra*, nine are from testis cDNA libraries, and one is from a larval fat-body library. We further investigated the pattern of expression of *hydra* in D. melanogaster by in situ hybridization. We found that *hydra* is expressed most intensely in the proximal region of the testis (Figure 5), where mature sperm are formed, suggesting that *hydra* functions in late stages of sperm development.

### Functional Consequences of the Evolving Gene Structure of *hydra*

To study the impact of exon 1 duplication on gene expression, we asked if each of the duplicated exons could function as a transcriptional start site. To determine whether each putative exon 1 is functional in *D. melanogaster,* we needed to use a technique that could distinguish these very similar potential transcripts. We therefore performed 5′ RACE analysis using RNA extracted from adult male testes of D. melanogaster and analyzed the sequences of 58 clones. The previously described Ra and Rb transcripts differ by three nucleotides at positions −21 to −23 in their 5′ UTRs as well as by a nonsynonymous polymorphism in their second codon. We designate alternative first exons 1, 2, 3, and 5 as class A (similar to Ra), and alternative first exons 4, 6, 7, 8, and 9 as class B (similar to Rb).

Some of these sequences can be further distinguished by additional polymorphisms further 5′ in the 5′ UTRs. Six different groups of alternative transcripts could be potentially distinguished, with two of these groups containing two or three identical first exons (Table 1). Among our 58 5′ RACE clones, four out of six of these groups were represented at least twice, demonstrating that at least four different alternative first exons in *hydra* are functional.

We also analyzed to see if each duplicated first exon contains core promoter sequences known to be important for the initiation of transcription of *Drosophila* genes [43]. We looked specifically for the TFIIB recognition element (−37 to −32 bp, GGGCGCC or CCACGCC), TATA box (−31to −26 bp; TATAAA); initiator (−1 to +4 bp; TCA(G/T)T(T/C)), and downstream promoter element (+28 to +32 bp; (A/G)G(A/T)(G/T)(G/A/C)). We found that almost all duplicated exon 1s contain all four core promoter sequences, with two exceptions: the sixth and the ninth duplicated exon 1s do not contain the downstream promoter element and the TATA box, respectively. Since both of these exons are used (Table 1), our results suggest that these two elements are not essential for transcription of *hydra.*

Using the sequences of the clones from 5′ RACE, we also mapped the sites for transcription initiation. We found that most transcripts of *hydra* do not use a fixed site for transcription initiation. Instead, “slippery promoters” were found to be used in different exon 1s, as described for other *Drosophila* genes by Yasuhara et al. [44].

**Figure 6:**
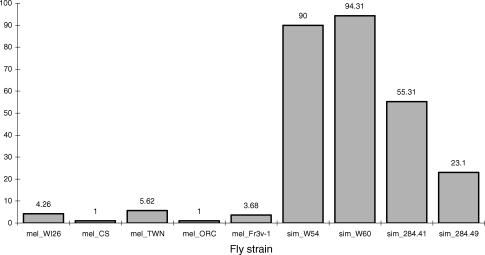
Relative Abundance of *hydra* mRNA Quantified by Real-Time RT-PCR in Various Lines of D. melanogaster and D. simulans For each line, the abundance of *hydra* relative to *GAPDH* was determined. This value was then compared for each line relative to D. melanogaster Canton S (mel_CS), which is normalized here to a value of 1.

We also tested whether *hydra* structural evolution might lead to expression-level differences between sibling species by quantifying *hydra* expression levels in five wild lines of D. melanogaster and in four wild lines of D. simulans (Figure 6). Strikingly, *hydra* was expressed at a substantially higher level in all D. simulans lines compared with all D. melanogaster lines. Among all pair-wise comparisons, the fold increase in D. simulans ranged from ~4 to ~150, with the mean fold difference being ~22. These data indicate that *hydra* has lower expression in *D. melanogaster,* despite having multiple alternative transcription start sites.

### Nonneutral Evolution of *hydra*

*hydra* contains an intact reading frame in all *melanogaster* subgroup species, encoding a predicted protein of 302, 280, and 308 amino acids in *D. melanogaster, D. simulans,* and *D. yakuba,* respectively. We calculated Ka/Ks ratios of the coding region of *hydra* among species in the *melanogaster* subgroup (Table 2). The ratio is <1 in all comparisons, suggesting that *hydra* is evolving under some degree of functional constraint.

**Table 2:**
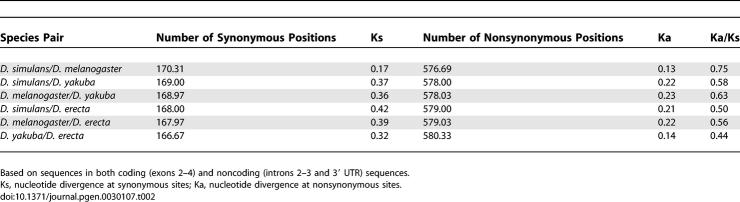
Summary of Ka and Ks Analysis of *hydra* among Four Species of the *melanogaster* Subgroup

To gain further insight into the evolution of *hydra,* we collected population samples from *D. melanogaster* and *D. simulans,* which have the highest interspecific Ka/Ks ratio of 0.75 (Texts S9 and S10). We found that the nucleotide diversity at both synonymous (πs) and nonsynonymous (πa) sites in D. melanogaster is similar to the average of other functional genes, with about ten times more polymorphism at synonymous sites relative to nonsynonymous sites (Table 3). In contrast, there is not much difference between πs and πa in *D. simulans,* where πs is much lower than the average of other genes for this species [45]. In order to further investigate whether this unusual pattern of nucleotide diversity (similar levels of synonymous and nonsynonymous polymorphism) in D. simulans is lineage specific, we studied polymorphism in *D. yakuba.* πs and πa of *hydra* in D. yakuba are similar to those of *D. melanogaster,* and πs is similar to the average value (0.0127) of six X-linked loci in D. yakuba [46], suggesting that the unusual pattern of nucleotide diversity of *hydra* is restricted only to *D. simulans.* The approximately equal values of πs and πa in D. simulans may suggest that *hydra* is under reduced functional constraint in *D. simulans.* Two findings suggest, however, that *hydra* is not a pseudogene in *D. simulans.* First, it has an intact open reading frame, and second, *hydra* is expressed in D. simulans and has a similar expression pattern to other species (see above).

**Table 3:**
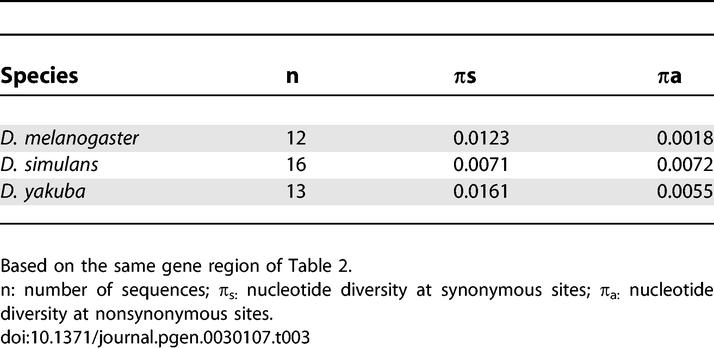
Summary of Polymorphism Data from *hydra* in D. melanogaster, *D. simulans,* and D. yakuba

Results of the McDonald-Kreitman (MK) test [47] among these three species are shown in Table 4. Neutral evolution of *hydra* can be rejected between D. melanogaster and *D. yakuba,* which we suggest is caused by the large number of nonsynonymous substitutions between these species. There is also a large amount of nonsynonymous divergence between D. melanogaster and *D. simulans.* We do not, however, reject neutrality in this case, likely due to the unusually low πs in D. simulans discussed above (Table 3). Thus, these results suggest that D. melanogaster is the most likely lineage in which *hydra* has experienced positive selection.

**Table 4:**
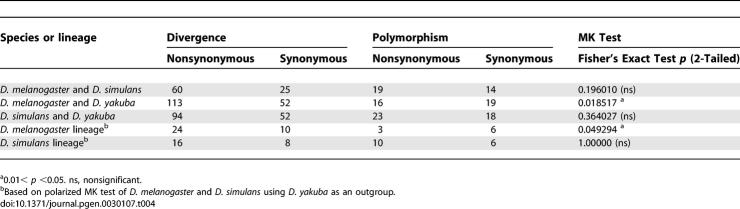
MK Tests for Nonneutral Evolution of *hydra*

To confirm this conclusion, we tested to see if the nonneutral evolution of *hydra* occurred in a lineage-specific pattern by performing polarized substitution analysis with the MK test (Table 4). Replacements were polarized to each species lineage using *D. yakuba hydra* as an outgroup sequence. The polarized MK test is significant only in the D. melanogaster lineage.

## Discussion

### *hydra* Is a Recently Originated Functional Gene

We identified the gene *hydra* in *D. melanogaster,* but could only identify orthologs within other *melanogaster* subgroup species. Using both BLAST searches of full-genome sequences with the 6-kb region flanking *hydra* and Southern hybridization, we were unable to identify *hydra* from *D. ananassae, D. pseudoobscura,* or *D. virilis.* We were, however, able to identify the syntenic region in these species (Figure 1B). These data strongly suggest that *hydra* originated in the common ancestor of the *melanogaster* subgroup.

All available evidence also suggests that *hydra* is a functional gene. First, all *melanogaster* subgroup species contain intact open reading frames with no stop codons, even though there are many insertions and deletions in the *hydra* CDS among species. Second, Ka/Ks values between all species are less than 1, suggesting that *hydra* is under functional constraint (Table 2). Third, the level of polymorphism of *hydra* in D. melanogaster is similar to known functionally important genes (Table 3). Fourth, RT-PCR and in situ hybridization show that *hydra* is predominantly expressed in male testes (Figures 4 and 5).

### *hydra* Shares Common Features of New-Gene Evolution

Several other cases of new-gene evolution in *Drosophila* have been studied [2,48]. Examples include (1) *Jingwei,* a chimeric gene that evolved as a result of the insertion of an *Adh* retrosequence into a duplicated locus in the *D. teissieri/D. yakuba* lineage [19]; (2) *Sdic,* which originated through a complex set of rearrangements, including a gene fusion between the gene encoding the cell adhesion protein annexin X and a cytoplasmic dynein intermediate chain (*Sdic* encodes a novel sperm-specific dynein found only in D. melanogaster) [49]; (3) *Sphinx,* a recently evolved gene apparently created by both retroposition and exon shuffling, which produces a novel noncoding RNA gene found only in D. melanogaster [33]; (4) *mkg,* a gene family generated originally as retroposed duplicates about 1–2 million y ago that further evolved by individual members undergoing gene fission and fusion events [35]; (5) *K81,* a gene that arose through duplication by retroposition after the radiation of the *melanogaster* subgroup and acquired a new male germline–specific function [50]; (6) *Dntf-2r,* a rapidly evolving male-specific gene in D. melanogaster and sibling species, which originated from retroposition [34]; and (7) five protein-coding genes which appear to have originated in D. melanogaster from noncoding DNA and evolved male-biased expression [6].

Despite the diverse mechanisms involved in the creation of these young genes in *Drosophila,* there are two features common to most of them: (1) they evolve rapidly in sequence, including at nonsynonymous sites for protein-coding genes [19,20,34,49,51,52]; and (2) their expression is often male-specific, and in many cases testis-specific [6,52]. This expression pattern was also often found in a large survey of new retroposed genes [17].

*hydra* shares both of these characteristics. The Ka and Ks values between D. melanogaster and *D. simulans* were substantially higher than the average values of most other genes, and the Ka/Ks ratio ranged from 0.44 to 0.75 in all species compared (Table 2). These high values may be explained by accelerated functional divergence following the origin of new genes. However, the elevated Ka/Ks ratio could also result from an elevated mutation rate and reduced selective constraint. To further investigate the evolution of *hydra,* we analyzed the level of polymorphism and divergence among *D. melanogaster, D. simulans,* and *D. yakuba.* Our population genetics analyses rejected the null hypothesis of neutral evolution in D. melanogaster (Table 4). This rejection appears to be due to a significant excess of fixed differences at nonsynonymous sites in *D. melanogaster,* suggesting that positive selection has driven the evolution of *hydra* in *D. melanogaster.*

### *hydra* Is Expressed in the Basal Part of the Testis

The male-specific expression pattern is a second feature that *hydra* shares with other new genes. For many newly evolved genes, male-specific or testis-specific expression has been demonstrated by RT-PCR, but the spatial expression patterns are unknown, making it difficult to refine our knowledge of what aspects of testis development or spermatogenesis may be under selection. We determined that *hydra* is expressed most intensely in the basal (proximal) part of the testis in D. melanogaster by in situ hybridization (Figure 5). This localization pattern suggests that *hydra* may have a function in late-stage spermatogenesis or sperm differentiation. We note that *hydra* is X-linked, which suggests that it escapes from the X inactivation that occurs during spermatogenesis [53]. Our results also stand in contrast to results from genome-wide studies that suggest that genes with male-biased expression as well as newly evolved testis-specific genes tend to be autosomal [17,54,55]. Interestingly, the newly evolved testis-expressed genes *Sdic* and *CG15323* are also located in polytene region 19 of the X chromosome [6,49].

Several studies have shown that genes with male-biased expression have significantly faster rates of evolution than genes with female-biased or unbiased expression [56,57]. This difference is caused primarily by a higher Ka in the male-biased genes. Sexual selection is likely to be driving this acceleration of male-biased genes, but functional tests will be required to understand fully the role of selection. Our study of *hydra* suggests that late-stage spermatogenesis may be the key developmental process to investigate further for functional differences between species.

### Novel Aspects of *hydra: hydra* Has Undergone Recurrent Structural Evolution

Our analysis of *hydra* reveals several aspects that are unusual or unique compared with other newly evolved genes. One novel feature of *hydra* is that it has experienced recurrent structural evolution in the lineages leading to both D. melanogaster and D. simulans (Figure 1). We can infer the likely occurrence of three independent structural changes to *hydra.* One is a duplication of exon 1 in the common ancestor of D. simulans and *D. sechellia.* Second is a duplication of the whole locus in D. simulans and *D. sechellia.* Although uncertainties remain about the structure of the *hydra* region in *D. sechellia,* our phylogenetic analyses suggest that *hydra* duplicated independently in these two species. Third is the further duplication of seven additional exon 1s in *D. melanogaster.* Our phylogenetic analysis suggests that these additional duplication events occurred only in *D. melanogaster,* rather than in the common ancestor of *D. melanogaster, D. simulans,* and *D. sechellia,* followed by loss in the latter two species. It is possible, however, that the phylogenetic signal could be erroneous if homogenization occurred by continual gene conversion and/or unequal crossing over among these short duplicated exons.

The duplicated exon 1 in D. simulans and D. sechellia and most of the additional exon 1 duplications in D. melanogaster are flanked by partial *DINE-1* elements. *DINE-1* is not associated with *hydra* in D. yakuba or *D. erecta.* Genome-wide analyses of *DINE-1* suggest that *DINE-1* was transpositionally active and then silenced in the common ancestor of D. melanogaster and *D. yakuba,* followed by another round of activity and silencing in D. yakuba [39]. These findings would suggest then that *DINE-1* inserted ancestrally in *hydra,* followed by loss in the D. yakuba lineage but not the D. melanogaster lineage. An alternative scenario is that *DINE-1* may have inserted in *hydra* during a period of activity in the *D. melanogaster/D. simulans* ancestor after divergence from D. yakuba that was too brief to have a left a genome-wide footprint.

Regardless of the timing of the initial *DINE-1* insertion into *hydra,* the further duplications of exon 1 in D. melanogaster were unlikely to have occurred by *DINE-1* transposition because alternative exon 1s two through eight contain UTR and CDSs that are adjacent to rather than within the *DINE-1* sequences. As noted above, the phylogenetic relationships among these alternative exons 1s suggest that they originated by unequal crossing over. *DINE-1* may have promoted these duplications by increasing the homology among exon 1s. While we lack evidence for a direct role of *DINE-1* in driving *hydra* structural evolution, our results do suggest that a property of transposable elements that is usually considered to be deleterious—their ability to cause ectopic recombination and unequal crossing over—may also contribute to structural evolution of genes. We have observed other cases of structural variation associated with *DINE-1* insertions in the *melanogaster* species complex (unpublished data). For most genes, evolving under strong functional constraint, a *DINE-1* insertion that causes structural changes in the locus is likely to be highly deleterious. However, for a newly evolved gene like *hydra,* there may be more flexibility in terms of tolerating gene structure changes. We also suggest that as a gene evolves in structure, it may accelerate the rate of CDS evolution.

More generally, our evidence that *hydra* has undergone multiple and independent structural changes suggests that gene structure as well as CDSs may evolve rapidly in new genes, even without any association with transposable elements. One other example is the *monkey king* gene family in *Drosophila,* which has undergone rapid structural changes caused by gene fission [35].

### *hydra* Expression Level Is Evolving

A second novel aspect compared with other studies of newly evolved genes is that *hydra* expression level has dramatically changed between sibling species. Our quantitative RT-PCR results showed that the expression level of *hydra* between D. melanogaster and D. simulans is highly different (Figure 6). *hydra* in D. melanogaster is expressed at least 20-fold less on average than in *D. simulans.* Why is the expression level so different between these two closely related species?

Two hypotheses may explain the decreased *hydra* expression in *D. melanogaster.* First, it may be caused by competition or interference among the tandemly duplicated heads during the transcription initiation stage. In this model, incomplete transcription initiation complexes would form at multiple heads. Reduced transcription initiation could result from either sequestration of transcription factors or direct interference among promoters. Second, it may be the result of differences in the degree of heterochromatinization of the *hydra* region between D. melanogaster and *D. simulans. hydra* is located near the pericentromeric region of the X chromosome. This region may be under continuous pressure from encroachment of the nearby heterochromatic pericentromere, leading to species-specific differences in chromatin states. Increased numbers of TE insertions are one characteristic of heterochromatic regions, and the large number of *DINE-1* insertions in D. melanogaster is consistent with the hypothesis that the *hydra* region is partially heterochromatic.

Our views on the evolution of genome structure and function have changed dramatically in the past decade as more whole-genome sequences have become available in various species. Repetitive elements are a major cause of structural changes and instability in genomes. At the cytological level, chromosome rearrangements often occur at repetitive sites [31,58,59]. Our identification of the *hydra* gene suggests that repetitive elements also contribute to the formation and evolution of new genes by causing structural changes in gene organization, driving the evolution of new exons and transcription start sites, and promoting divergence in gene expression.

### *hydra* Is a Rare Example of De Novo Gene Creation

One of our most striking findings is that *hydra* appears to have been created de novo rather than being a duplicate of a pre-existing gene. Two pieces of evidence support this hypothesis. First, *hydra* (and *CG1835*) are found only in *melanogaster* subgroup species. The surrounding ~26 kb of DNA is found in other *Drosophila* species that lack *hydra* (Figure 1B). Second, *hydra* shares no homology with any known or predicted proteins. Arguing against this hypothesis of de novo origin is the fact that all forms of *hydra* contain four predicted exons. One might expect that a recently evolved de novo gene would have a simpler one-exon structure, as has been observed for several candidate de novo genes in D. melanogaster [6]. Nevertheless, if *hydra* does have an ortholog in *Drosophila* outside of the *melanogaster* subgroup, it must have evolved so rapidly as to share no detectable similarity with any predicted proteins, including in multiple *Drosophila* genomes or other insect genomes that have been completely sequenced. If there is a highly diverged *hydra* parental gene or ortholog in other *Drosophila,* it also must have transposed to a novel genomic location in the *melanogaster* subgroup. Considering all the available data, we suggest instead that *hydra* evolved de novo from DNA sequence that inserted between *run* and *cyp6v1* in the common ancestor of the *melanogaster* subgroup.

### The Dynamic Nature of New Genes

The most common fate of newly formed genes is disappearance. For example, about 85% of the genes duplicated during the yeast whole-genome duplication have been lost in Saccharomyces cerevisiae [3,60]. New genes therefore can be viewed as analogous to mutations, with most being neutral or deleterious in effect and therefore subject to elimination. A common characteristic of successful new exons and new genes is a high rate of CDS evolution [7,20], and *hydra* shows this evolutionary pattern. Our discovery of *hydra* suggests that gene expression level and gene structure may also evolve rapidly and recurrently in new genes. We suggest that the successful retention of new genes requires maximal variation.

## Materials and Methods

### Fly strains.

Flies used in this study include: (1) isofemale lines of D. melanogaster (WI) from Sergey Nuzhdin (University of California Davis, Winters, California, United States); (2) strains of D. melanogaster from worldwide populations: California (CA), Canton S (CS), Taiwan (TWN), Oregon R (ORC), France 3V-1 (Fr3V-1) (gift of S. C. Tsaur, Academia Sinica, Taiwan); (3) isofemale lines of D. simulans from African populations (mad, sz, su, zk lines; gift of John Pool, Cornell University, Ithaca, New York, United States), and one line each from France (W54) and Japan (W60); (4) isofemale lines of D. yakuba (cy lines) from Africa (gift of Peter Andolfatto, University of California San Diego, United States); and (5) strains of *Drosophila* species, including *D. yakuba* (Tai18E2, and 286.82–90), *D. erecta, D. ananassae, D. virilis,* and D. pseudoobscura (gift of S. C. Tsaur).

### DNA preparation and analysis of DNA sequences.

DNA was extracted from pools of ~50 male flies from each line by a standard phenol-chloroform extraction followed by ethanol precipitation. Primers to amplify and sequence the *hydra* gene regions from *D. melanogaster, D*. *simulans,* and D. yakuba were designed using the homologous sequences obtained from BLAST searches against Flybase genome databases (http://www.flybase.org/blast). Primers for both D. melanogaster and D. simulans are IN1_F (5′-GCGTTTGTACACTTGGCAAC) and 3UR (5′-TGATGTAGGAATATGCGTTGC), and for D. yakuba are yakE2_F (5′-CTTCAGAGAACCCCAACCAA) and yakE3_R (5′-CTTGCAATTATCCGGATTGC). In *D. simulans,* the primer pair specifically amplifies the second duplicate of *hydra,* which is adjacent to the S3 alternative exon 1 (Figure 1B). Other primer sequences and PCR amplification conditions are available upon request. PCR products were directly sequenced in both direction using ABI BigDye (Applied Biosystems, http://www.appliedbiosystems.com) technologies under slight modification of the manufacturer's suggested protocols. All alleles were sequenced on both strands. Sequences are deposited in GenBank (http://www.ncbi.nlm.nih.gov/Genbank) under accession numbers EF596837–EF596877.

### Southern hybridization.

Genomic DNA was extracted from 60–70 flies of each line with phenol-chloroform, was ethanol precipitated, and was diluted to a final concentration of about 5 μg/μl. A total of 15 μg DNA was then digested separately with SacII, separated on a 0.8% agarose gel, and transferred to a nylon membrane by Southern blotting. Probes were from PCR product using DNA template from D. melanogaster with primers hydra_rep500_R2 (5′-CAGT(T/C)AGACCTCTCTGAAATC) and E2_146R (5′- GGTTGGAATCCTCTGGAGTGTTGG), and were prepared by DIG-labeling Nick translation kit (Roche, http://www.roche.com). Hybridizations were done at 50 °C.

### RNA preparation, RT-PCR, 5′- RACE, and molecular cloning.

Testes of adult males aged 2–5 d old were dissected in PBS buffer and immediately transferred to PBS buffer on ice. RNAs were extracted with Trizol (Invitrogen, http://www.invitrogen.com). After a brief vortex mixing, the mixture was incubated at 4 °C for 15 min and then centrifuged at low speed to obtain phase separation. The aqueous phase was transferred to a fresh tube, to which an equal volume of isopropanol was added in the presence of 3 μl glycogen. The mixture was kept at −20 °C for 30 min, followed by centrifugation. The RNA pellet thus obtained was washed with 70% chilled ethanol, repelleted, and air dried. The final RNA pellet was dissolved in 20 μl DEPC-treated distilled water and was used in at least two RT reactions.

RT was performed using the Superscript II RT kit obtained from Life Technologies (http://www.invitrogen.com) using oligo(dT)_12–18_ as the primer. The RT products were dissolved in 20 μl sterile distilled water and 1 μl was used for each PCR. For 5′-RACE), first strand synthesis using the lock-docking oligo(dT) primer and 35 cycles of PCR amplification were performed using the SMART RACE cDNA Amplification Kit purchased from Clontech (http://www.clontech.com). The *hydra*-specific primers used in this study are E3_110R (5′-TGGATTTTACGCCGCTCCT). 5′ RACE–derived PCR products were subcloned into the pGEM-T vector (Promega, http://www.promega.com) using a standard cloning procedure, and 61 clones were picked randomly for DNA sequencing.

### Quantitative PCR.

A total of 50 testes of each line were collected from 3-d-old males. Testes were homogenized in Trizol (Invitrogen), and total RNA was phenol-chloroform extracted, ethanol precipitated, and cleaned up with RNeasy mini kit (Qiagen, http://www.qiagen.com) and RNase free DNase Set (Qiagen). About 200 ng of total RNA was used for synthesizing cDNA with TaqMan Reverse Transcription Reagents (Applied Biosystems) using oligo-dT as the primer, following the manufacturer's instruction.

Quantitative real-time PCR (qPCR) was performed using the Lightcycler-FastStart DNA Master SYBR Green I kit (Roche), according to the manufacturer's instructions. Primers used were E2_222F (5′-ATGTGGCAAAGGTCCAGAAT) and E3_110R (5′-TGGATTTTACGCCGCTCCT), which are complementary to exon 2 and 3 of *hydra,* respectively. Note that this primer pair cannot distinguish transcripts derived from the two duplicates of *hydra* in *D. simulans.* PCR quality and specificity was verified by melting curve dissociation analysis and gel electrophoresis of the amplified products. Relative transcript abundance of *hydra* was calculated using the second derivative maximum values from the linear regression of cycle number versus log concentration of the amplified gene. Amplification of the control gene *GADPH* was used for normalization. Sequences of the primers used are GADPH_F: 5′-AAGGGAATCCTGGGCTACAC and GADPH_R: 5′-CGGTTGGAGTAACCGAACTC.

### In situ hybridization.

Digoxigenin-labeled antisense and sense control riboprobes were generated from a cDNA containing the sequence of exon 2 and 3 of *hydra* cloned into a pBluescript vector. Probes were synthesized using DIG RNA labeling mix (Roche), followed by treatment with 2 U DNAse (Promega) and incubation in carbonate buffer for 40 min at 65 °C as described in [61]. Probes were then ethanol precipitated, air dried, and resuspended in 200 μl hybridization buffer. Probes were used at a dilution of 1:50 during hybridization.

Testes were dissected in 1× PBS and kept on ice until fixation. Two fixations were done with 4% formaldehyde in 1× PBS for 20 min on ice. After washing twice in PBS/0.1% Triton-X, protease treatment and all following steps were done as described in [61]. Hybridizations were done at 60 °C overnight.

### Identification of *hydra* orthologs.

Orthologs of *hydra* were from the following prepublication genome sequences: D. simulans and D. yakuba sequence from the Washington University Genome Sequencing Center (http://genome.wustl.edu); D. sechellia sequence from the Broad Institute (http://www.broad.mit.edu); and D. erecta sequence from Agencourt Bioscience (http://www.agencourt.com). Sequences of *hydra* from these species were annotated by maximizing homology to the D. melanogaster sequence, and are shown in Texts S1–S6.

### Sequence analysis.

Sequences were aligned using ClustalW [62], with some manual adjustment to keep gaps in-frame in coding regions. Phylogenetic analyses were performed using Mega 3.1 [63]. Sequence polymorphism and divergence analysis were done using DnaSP 4.10 [64]. The effective number of synonymous and nonsynonymous sites, Ka and Ks values, and pairwise divergence for synonymous sites based on Kimura's two-parameter model were estimated.

## Supporting Information

Text S1 Annotation in GenBank Format of *hydra* Region Sequences in D. melanogaster
(54 KB TXT)

Text S2 Annotation in GenBank Format of *hydra* Region Sequences in D. simulans
(71 KB TXT)

Text S3 Annotation in GenBank Format of *hydra* Region Sequences in *D. sechellia scaf 8*
(42 KB TXT)

Text S4 Annotation in GenBank Format of *hydra* Region Sequences in *D. sechellia scaf 600*
(13 KB TXT)

Text S5 Annotation in GenBank Format of *hydra* Region Sequences in D. yakuba
(43 KB TXT)

Text S6 Annotation in GenBank Format of *hydra* Region Sequences in D. erecta
(43 KB TXT)

Text S7 Alignments in FASTA Format of *hydra* Exons 2–4 Used for the Phylogenetic Analysis in Figure 3
(7 KB TXT)

Text S8 Alignments in FASTA Format of *hydra* Exon 1 (Including Predicted 5′ UTRs) Used for the Phylogenetic Analysis in Figure 3
(5 KB TXT)

Text S9 Alignment of All DNA Sequences Used in the Polymorphism and Divergence Analyses (84 KB TXT)

Text S10 Alignment of Amino Acid Sequences Based on the DNA Alignment in Text S9
(12 KB TXT)

## Abbreviations

CDS: coding sequence
*DINE-1*: *Drosophila* interspersed element-1
MK test: McDonald-Kreitman test
RACE: rapid amplification of cDNA ends
TE: transposable element
UTR: untranslated region
